# Diatoms constrain forensic burial timelines: case study with DB Cooper money

**DOI:** 10.1038/s41598-020-70015-z

**Published:** 2020-08-03

**Authors:** Thomas G. Kaye, Mark Meltzer

**Affiliations:** 1 Foundation for Scientific Advancement, Sierra Vista, Arizona, USA; 2 228 Byron St, Palo Alto, CA 94301 USA

**Keywords:** Limnology, Ecosystem ecology

## Abstract

Diatoms are found universally in waters around the world. Some diatom species such as *Asterionella formosa* have a broad variation in seasonal abundance leading to the possibility that diatoms could constrain the time of year when an object was immersed in water. Here we apply this technique to the cold case of DB Cooper’s money. Nine years after the crime, six thousand dollars in three bundles were found on the shore of the Columbia River near Portland, Oregon. This burial site was ~30 km from his reported jump location which gave no apparent reason for the money to end up there. This study found diatoms on a recovered bill which indicates that the money was immersed before burial. The species mix found on the bills was compared to a test bill submerged in the Columbia River in November which was the timeframe for the crime. The Cooper bill contained diatoms from summer bloom species suggesting that the money was not directly buried dry and the immersion happened months after the late November hijacking. This finding rules out of a majority of current theories related to the crime and proposes diatoms as a feasible methodology to constrain seasonal timelines in forensics.

## Introduction

Diatoms are photosynthetic eukaryotic algae found in waters around the world^1^. There are thousands of species but all are characterized by a silica shell called a frustule that surrounds the organism with extremely complex morphology^2^. This frustule is made of a biogenic opal-A^3^ microprecipitated by the organism essentially similar to glass^4^. These shells can also incorporate trace elements from the surrounding water that can change in proportion depending on availability throughout the year^5^. Forensically diatoms are most often used in the diagnosis of death from drowning, as opposed to death before immersion^6–8^. Their extended use in forensics has been underutilized^9^ with successful cases in other areas showing great potential^10^. These include connecting a suspect to particular aquatic location^11^ and diatoms transferred to clothing^9^.

**Figure 1:**
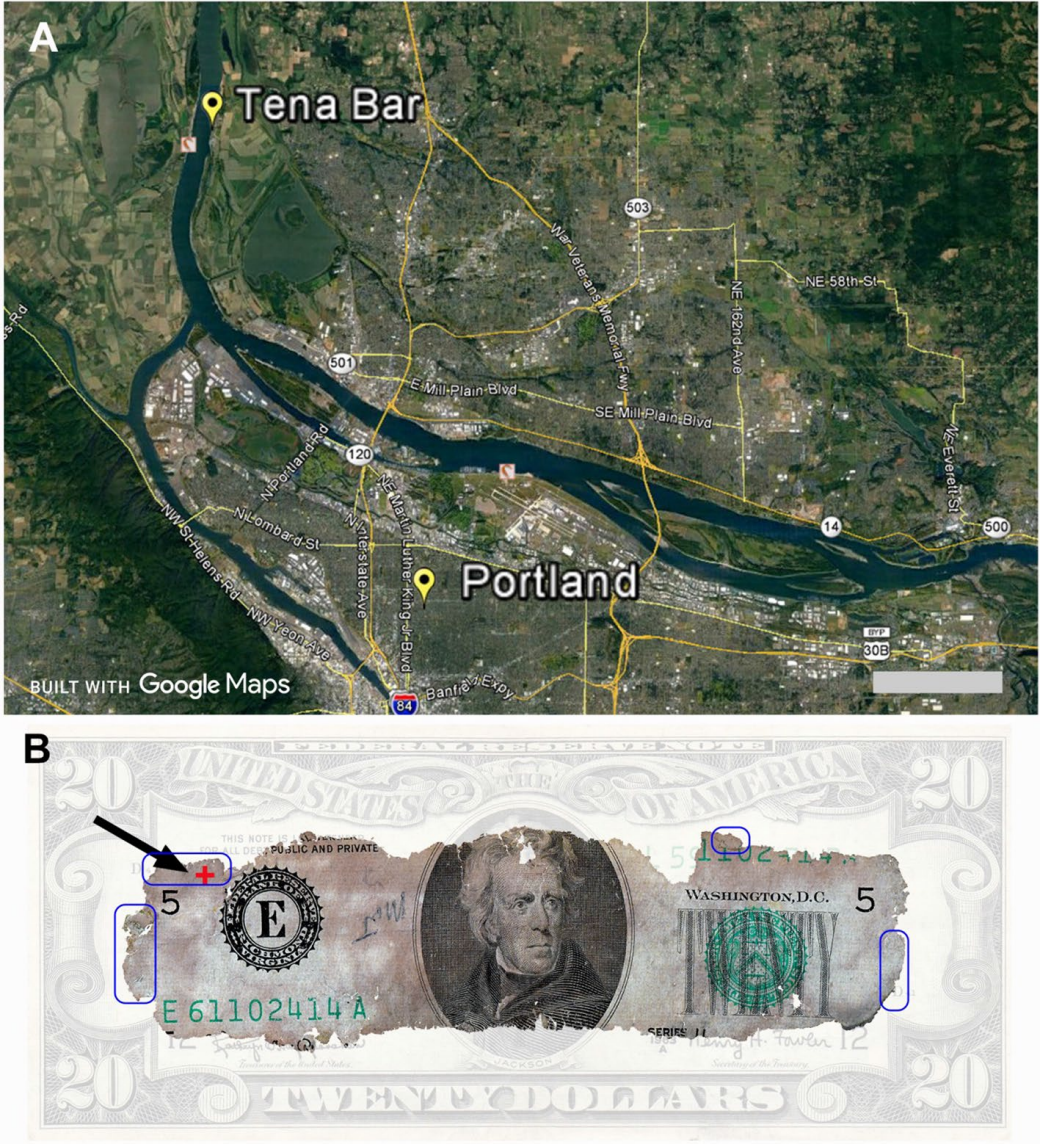
**(A)** Location of money find on the Columbia River beach known as Tena Bar north of Portland Oregon. **(B)** 20 dollar bill from the Cooper hijacking badly degraded around the edges. Blue boxes mark areas sampled for investigation. Red “+” marks location of *Fragilaria* diatom. Map drawn from Google Earth Pro software ver. 7.1.7.2606 Scale bar 5 km.

The DB Cooper skyjacking remains the only unsolved hijacking in US history^12^. The FBI closed the case in 2016 and since then freedom of information act requests have provided thousands of pages of internal 302 information from the FBI^13^. Since the time of the event on November 24th, 1971, there was no physical evidence found until nine years later on February 10th, 1980. At that time, a young boy named Brian Ingram, digging a fire pit on the shore of the Columbia River at Tena Bar (Fig. 1A), rolled up ~6,000 dollars in three bundles of twenty dollar bills with serial numbers matching the Cooper ransom (Fig. 1B). The rubber bands were intact but crumbled off and the bills were badly deteriorated around the edges^14^. The bundles were solid lumps that had to be separated professionally and authenticated later in a lab.

The Cooper money remains enigmatic because the beach burial location was ~30 km away from the reported jump zone over Ariel Washington. There was no evidence of a bag that would have kept the bundles together. At the time of the discovery, geologist Leonard Palmer investigated the site and proposed the “Washougal Washdown Theory”^14^ that proposed the money fell into a stream near Ariel and over a period of time, washed into the Columbia River upstream of the Tena Bar location where it was eventually found. Since then, a host of theories have been proposed for the money ending up on Tena Bar with no facts to constrain them.

Here diatoms are used to constrain the time of year leading up to the burial of the money on Tena Bar. It is believed to be the first time diatoms were used to constrain a seasonal timeline and hence this prototypical methodology has no history and will need to be verified in subsequent investigations. The burial along the Columbia was fortuitous since the river is historically well studied^15–17^ and its diatom species and abundances have been recorded on a monthly basis^18–20^.

## Methods

Diatoms make up 98% of the total plankton in the Columbia River^21^ and their frustules are robust providing a good target for analysis. For direct comparison, a diatom survey for genera and abundance were conducted on a single Cooper bill and two modern bills that were submerged in the Columbia River in March and November.

A single original Cooper bill owned by Brian Ingram was sampled for analysis. Sub-centimeter samples were removed (Fig. 1) and adhered to scanning electron microscope (SEM) stubs using double sided carbon tape. The stubs were gold coated in a vacuum evaporator. Five comparative samples were removed from the edges of two modern bills that had been soaked directly in the Columbia River for 15 min on November 13th which was seasonally 11 days before the skyjacking. A third set of samples was obtained from a 500 ml Columbia water sample taken on March 1st at the beach on Tena Bar. A modern bill was soaked in the water sample, air dried and four samples were placed on SEM stubs. Both modern bill samples were coated with palladium (Pd).

The samples were examined under a JEOL T300 SEM at various magnifications. Samples were raster scanned by eye at a magnification of 1,000x covering ~90% of the sample. Elemental analysis was done via energy dispersive spectroscopy (EDS) by an EDAX Octane Silicon Drift Detector at 10 and 15 kv. Spectra were analyzed with EDAX Team software. Diatom elemental spectra were normalized to the silicon peak for comparative analysis in Spectragryph software.

For testing diatom penetration into a buried stack of bills, two stacks of five modern bills were buried dry under ~9 kg of sand in a container that allowed flow through drainage. One stack was buried flat, the other vertical with a 2 cm sand layer over the top. Diatomaceous earth was mixed with water at 1 kg per liter which represented the maximum diatom density based on commercial production estimates^22^. Fifteen liters of diatom saturated water was filtered through the sand box and allowed to dry. Samples were then examined under the SEM looking for diatom penetration.

A 200 ml sand sample acquired at the surface of the money burial site on Tena Bar was processed for diatom extraction using a known protocol^23^. Hydrogen peroxide was added to moist sand with the addition of potassium dichromate and allowed to stand overnight. The sample was rinsed with distilled water, allowed to settle and decanted twice. Drops were allowed to evaporate on SEM stubs, coated in Pd and examined under the SEM.

One hundred modern bills wrapped in a rubber band were floated on water in an aquarium. The bundle was not agitated while the water infiltrated the stack. Over the course of minutes the stack fanned out and eventually sank to the bottom.

Diatoms were identified to the genus level. Species names are used here for clarity when they could be easily related to the published species list for the Columbia River.

## Results

**Figure 2:**
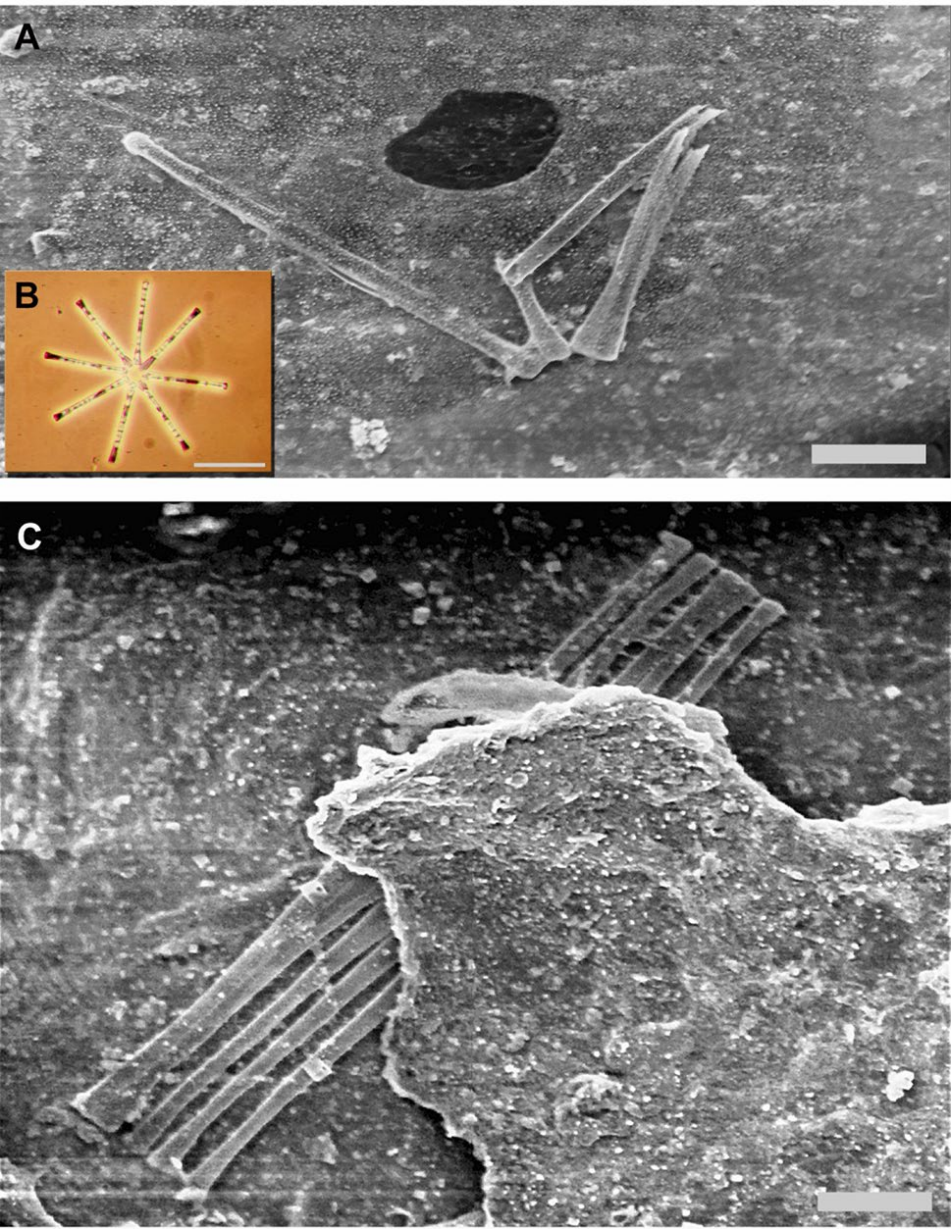
**(A)** Fractured but associated *Asterionella* found on the Cooper bill. Scale bar 10 µm. **(B)** *A. formosa* colony showing typical star structure. Scale bar 50 µm. **(C)** *Fragilaria* found sandwiched intact between two layers of bills demonstrating that this bill was in fact from the middle of the stack. Scale bar 10 µm.

The most notable result was that the genus mix for each sample had only small overlap. Table 1 lists the genera for each sample. Several examples of *Asterionella* were found on the Cooper bill. They are commonly found in star shaped colonies and live a planctonic lifestyle (Fig. 2B)^24^. They were found broken but associated suggesting that they were intact when they set down on the Cooper bill (Fig. 2A).

**Table 1:** Listing of diatom genera found on analyzed samples. Values reflect number of individual cells identified and normalized to quantity per square centimeter.

	*Asterionella*	*Fragilaria*	*Melosira*	*Stephanodiscus*	*Cyclotella*	*Cymbella*	*Navicula*
Season peak	May–June	September	All Year	July	Sept–April		
Cooper bill	16.10	2.98	0.60	1.79	1.79	1.19	–
November bill	0.23	1.36	0.23	–	0.45	–	0.68
March bill	–	–	0.28	–	–	–	–

A single *Fragilaria* was found intact and sandwiched between two layers of bills (Fig. 2C). Its position was 24 mm in from the edge of the stack when the bills were initially buried (Fig. 1B). It has similar spindle like morphology to *Asterionella* but this was the only one found intact.

**Figure 3:**
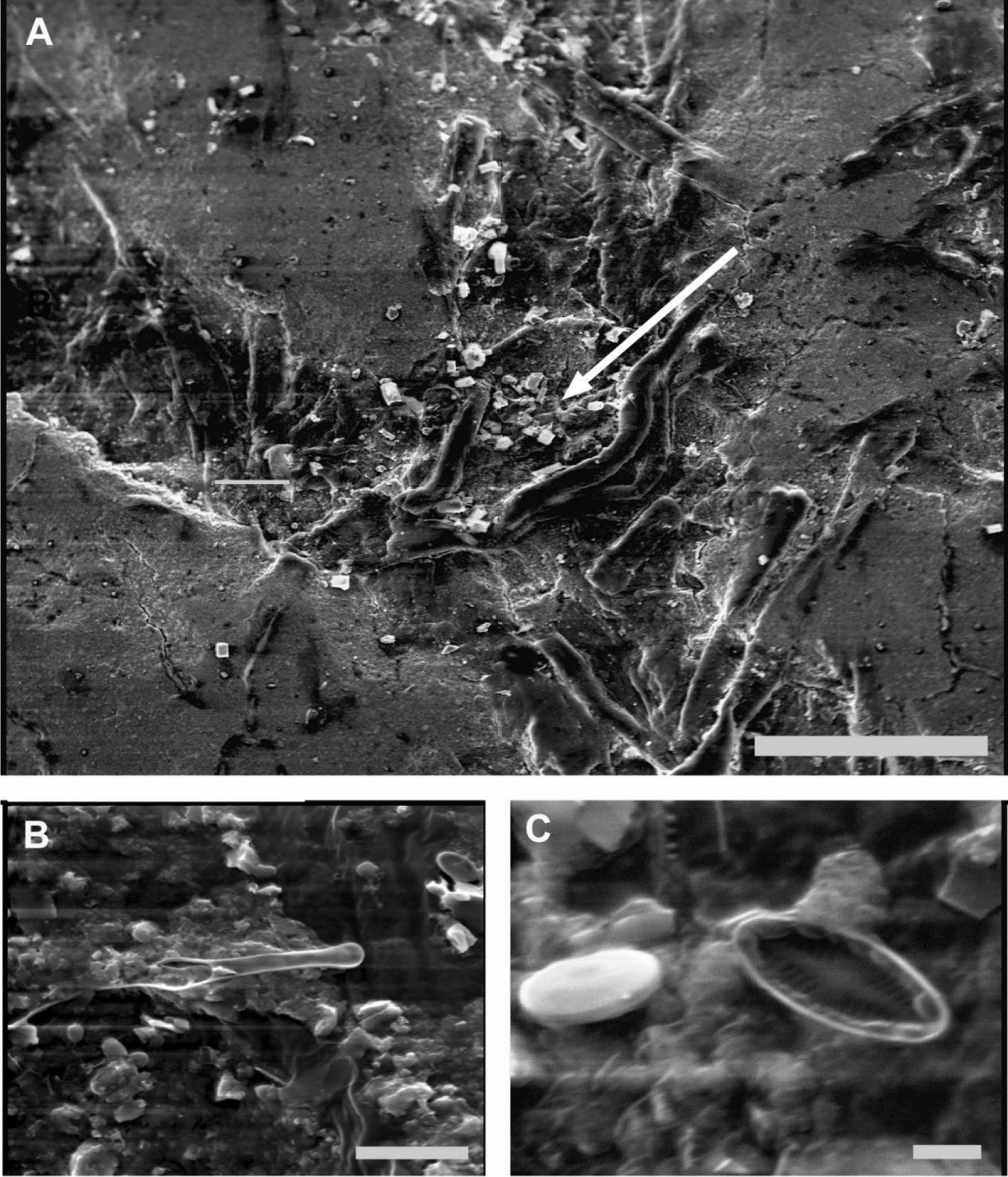
**(A)** Arrow points to build up of diatoms that penetrated the stack from the edge. The diatom buildup area is in the furrow between the printed ink visible as the smooth areas to the left and right. Diatoms were not found further inside the stack. Scale bar 100 µm. **(B)** Fragment found in the Tena Bar sand potentially from *Asterionella* or *Fragilaria*. Scale bar 10 µm. **(C)** small diatoms extracted from the Tena Bar sand sample. Scale bar 1 µm.

The diatom extraction from the surface sand at Tena Bar showed an abundance of smaller diatoms most below 5 µm in length (Fig. 3C). Genera in order of abundance, *Navicula, Cocconeis, Cymbell*a and *Nitzschia*. Only one fragment potentially from *Asterionella* or *Fragilaria* was found (Fig. 3B).

Selected specimens were analyzed in the SEM with EDS. Since matching genera were limited, a broad comparison was done across groups from each time frame. Spectra showed that there were differences between calcium and sodium in the two groups. The November specimens were slightly enriched with calcium and depleted in sodium, the Cooper bills were opposite (Fig. 6). A single diatom was found in the March sample and its spectrum had no enrichment of either sodium or calcium. There was one partial shaft of a suspected *Asterionella* or *Fragilaria* from the November Tena Bar sand that showed slightly increased sodium and calcium compared to *Asterionella* from the Cooper bill. This data is only qualitative since these small values are near the limit of EDS capability.

The bills buried under sand and then flooded with diatom saturated water did show some diatom infiltration from the edges (Fig. 3A). The Cooper ransom money was random used bills^14^, to accurately test diatom infiltration, used bills were selected in this experiment. The slightly rough texture of the bills created micro-voids at the edge of the stack where smaller diatoms like *Melosira spp*. as well as fragments could enter. Typically within 3–4 mm in from the edge, the void would close up due to the natural variation in texture and often a “tide line” was found where diatoms and fragments would pile up in larger numbers. Beyond the “tide line” the internal area of the stack was void of diatoms (Fig. 3A).

The money saturation experiment in the aquarium was performed to understand the reaction of a bill stack to water infiltration. The results demonstrated that the bills would sink after a short period of time and that the bills would fan out in the water. The individual surfaces of virtually all the bills were directly exposed to river water which would allow diatom infiltration.

## Discussion

The Cooper bundles were found just beneath the sand surface ~15 m up from the waterline. A sand slope angle of 10^∘^ was measured during a site investigation which would place the burial site ~3 m vertically above the water line. This location would only be immersed during times of high water and wave action. Dredging operations took place on the river and the sand was dumped slightly upstream of the burial location and could have contributed to additional sand on top of the bills. Sand is no longer deposited on the beach and it has undergone severe erosion. Rubber bands found intact but degraded on the bundles suggests they were initially buried without any significant exposure to the elements which is known to rapidly degrade them^25^.

In order to determine if a seasonal diatom timeline can be used to constrain the burial of the Cooper money, the first question to be answered is: can diatoms penetrate a bundle of money buried in sand? The diatom saturated water experiment showed that penetration is possible but only for the smaller range of diatoms and only a limited distance in from the edge on the order of millimeters. No “tide lines” of diatoms or small sand fragments were found on the Cooper bill. Since we know from the experiment that diatom accumulations were likely to happen on the edges, the lack of aggregations suggests they were destroyed with the severe degradation around the edges of the bundle. The inner degraded edge where the SEM samples were taken from showed no accumulations, suggesting the bills had congealed into a solid lump (consistent with the condition that the bills were found in), preventing any further diatom infiltration.

A second line of evidence that would signal diatom infiltration while buried would be an abundance of diatoms in the bills that were also found in the surrounding sand. The extraction of the diatoms from the Tena Bar sand showed a predominance of small forms on the order of 3–5 µm. These small diatoms are consistent with species that can survive in sand due to their ability to situate in the interstitial crevices of a single sand grain^26,27^. Larger diatoms, of which *Asterionella* and *Fragilaria* are among the largest, have low survivability in the proportionally boulder size sand grains^26^. The lack of predominantly smaller diatoms on the Cooper bill suggests little to no diatom infiltration to the inner portions of the stack occurred while buried. While similar small diatoms were found on the bills, they were not a dominant category as would be expected if they were the primary source of infiltration.

If the Cooper bill used in this examination was from the top of the stack, then one could expect to find a variety of diatoms from all sources. Figure 2C indicates conclusively that the examined bill is from the middle of the stack by finding an intact *Fragilaria* sandwiched between two bills. Due to the congealed nature of the bills, it was not uncommon to find intact fragments of other bills adhered to the larger bill. *Fragilaria* at ~80 µm^28^ is considered a larger diatom in the Columbia River system^29^. It is planktonic^30^ and therefore has no ability to move through sand. Its size and location interior to the stack (Fig. 1) and notably with no smaller diatoms surrounding it, suggests that it came to rest there while the bill was completely exposed to river water.

**Figure 4:**
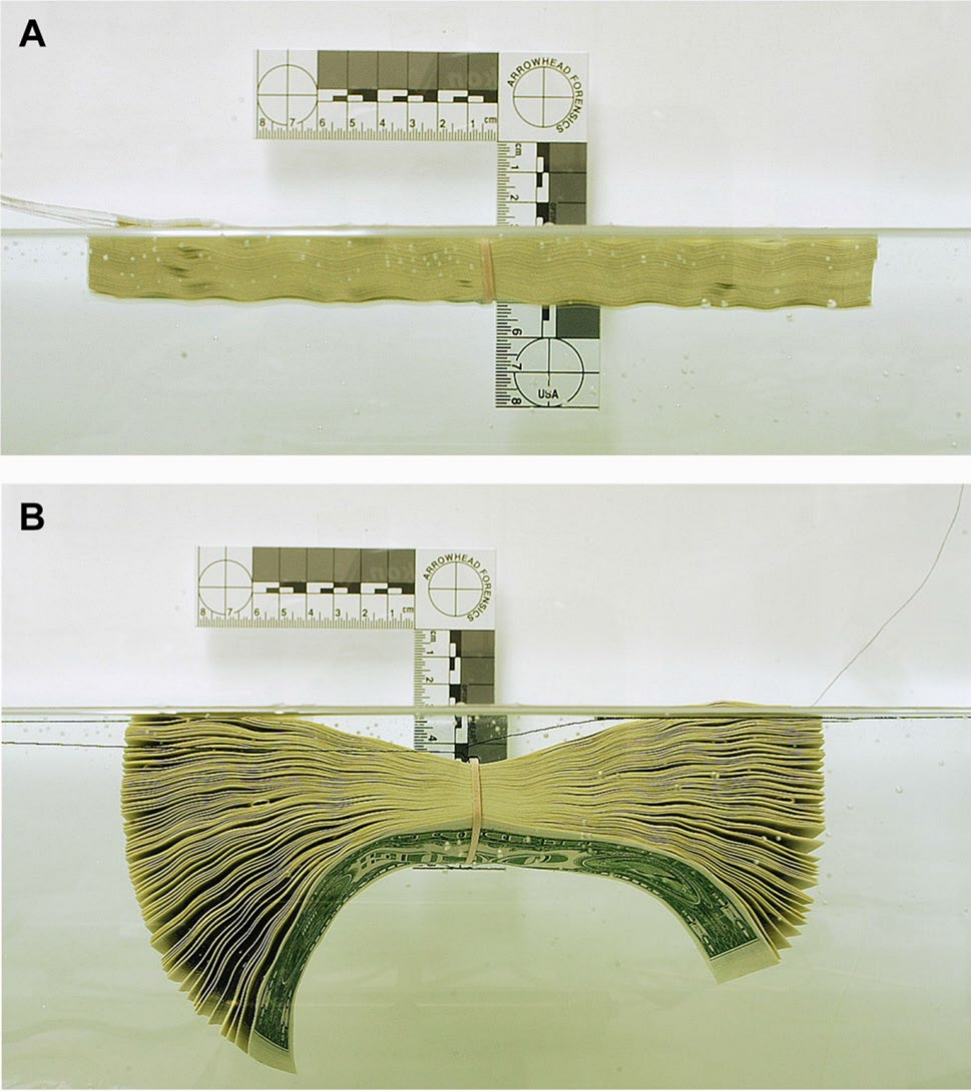
**(A)** Stack of bills bound with a rubber band immediately after placing in still water. **(B)** After several minutes, the stack becomes saturated and fans out exposing individual bills to the water. Shortly thereafter the entire stack will sink to the bottom.

If the previous experiments and investigations rule out diatom infiltration while buried, then the findings suggest that diatoms found their way onto the bills during water immersion. As shown in Fig. 4, a stack of bills once saturated, will fan out in water exposing all surfaces to micro-particles in the water environment. The exposure of the fanned out stack to the river, suggests the simplest way for large, intact but fragile diatoms to be found alone interior to the bill stack. This would have occurred prior to burial and be in the water long enough for fan out to occur.

The Columbia River has seasonal blooms of diatoms with different species found in winter vs summer^19^. If the bills were submerged for an extended period covering multiple seasons, then diatom species found on the bill should also represent multiple seasons. Table 1 shows the genera found on the Cooper bill and the dollar bill soaked in the Columbia in November. The first notable observation is that there is little overlap in genera between the two seasons.

**Figure 5:**
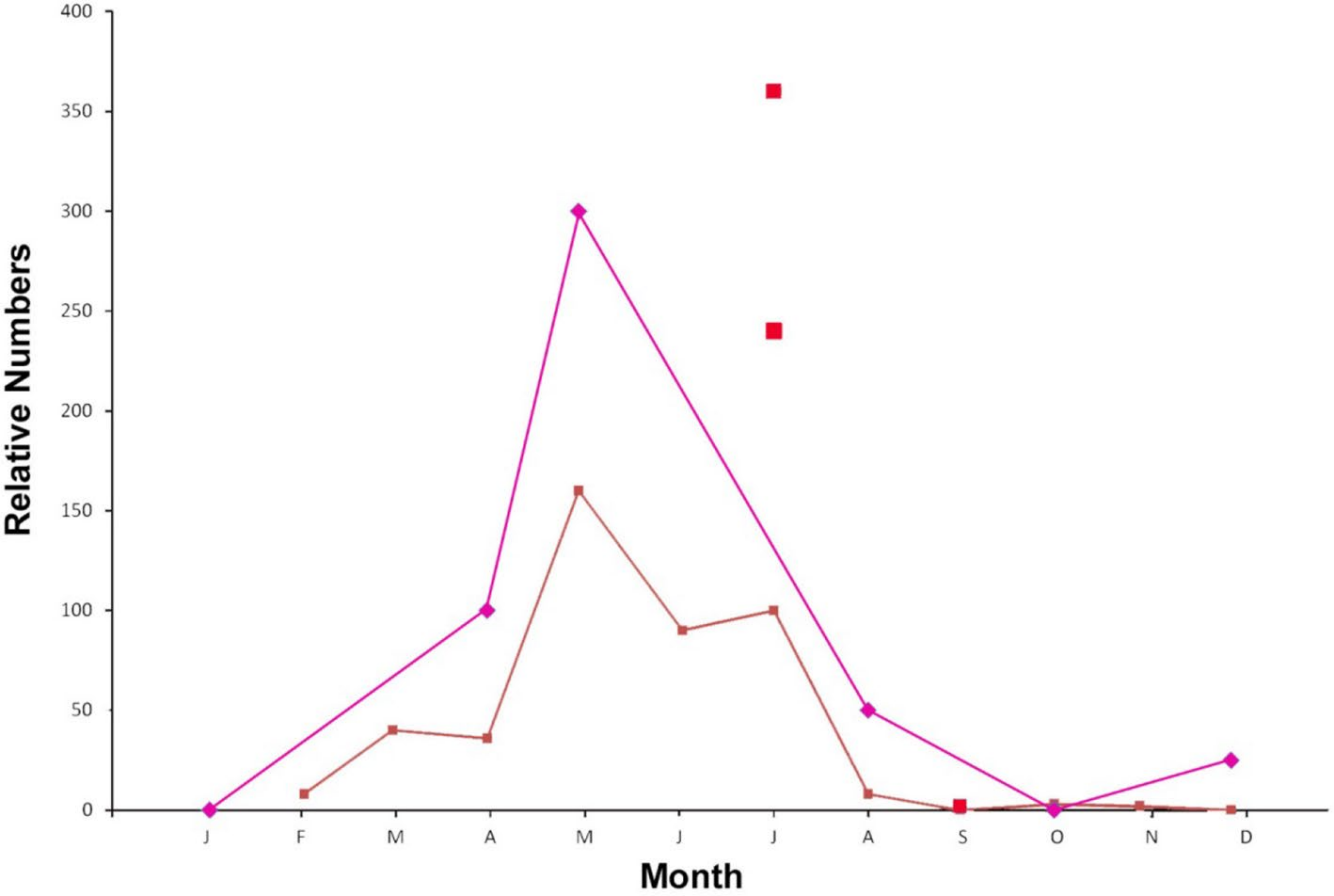
Monthly abundance of *Asterionella* showing population bloom in May and June. Extremely low numbers are apparent for winter months. Data compiled from three sources^19–21^ graph shows relative numbers.

*Asterionella* followed by *Fragilaria* are key indicators in this study. *Asterionella* are relatively large up to 100 µm^31^, planktonic diatoms that undergo radical changes in population in the Columbia River (Fig. 5) of up to 10 × during the course of the year^20^. They assemble into star shaped colonies that are susceptible to damage. *Asterionella* were found broken but associated on the Cooper bill as shown in Fig. 2A. Although in pieces, the relatively complete association of parts suggests that the diatoms landed intact on the bill and were subsequently crushed and broken after the fact. Similar associations were found elsewhere on the Cooper samples.

Several examples *Asterionella* were found on the Cooper bills and this diatom is nearly absent in November when the jump occurred^20,21^. There is however a very large bloom of *Asterionella* in early summer during the months of May and June^19,21^. The other diatoms identified on the Cooper bill such as Stephanodiscus are also more prevalent in the summer season^21^. The diatoms found on the November bill are not consistent with species found on the Cooper bill. This suggests that the Cooper bill was immersed during the summer *Asterionella* bloom and the length of submersion did not extend into subsequent seasons.

**Figure 6:**
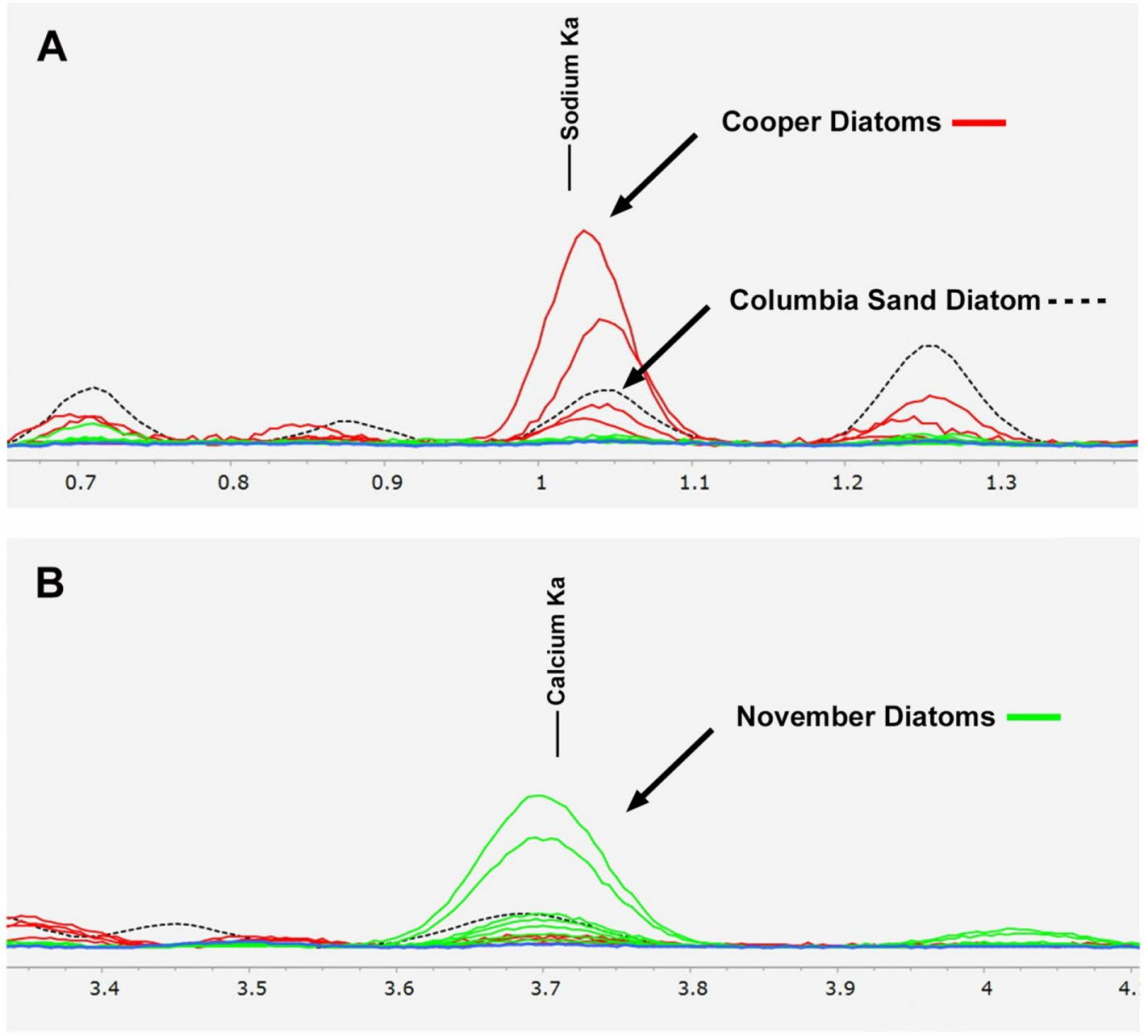
**(A)** EDS spectra overlay showing the sodium line. Red lines are spectra from the Cooper bill diatoms showing elevated sodium levels, green lines are from November samples. Blue line is the single *Asterionella* spectra from the November sand sample showing no enrichment in either sodium or calcium. **(B)** Calcium line showing elevated presence of calcium for November diatoms while Cooper samples show lower levels. Each group of diatoms showed opposite enrichment of sodium and calcium. Data is relative and qualitative.

Trace elements are incorporated into the diatom frustule during growth and elemental availability varies in rivers during the year^17^. Krivtsov et al. 2000 studied the elemental variation in *A. formosa* and found that it varied by the season^5^. There were not enough recovered *Asterionella* from the November time frame to do a direct comparison but elemental signatures from a variety of specimens were compared between the November and Cooper bills. Figure 6 shows the diatom’s elemental spectra of calcium and sodium overlaid. The spectra were normalized to silicon and show relative abundances. The detected levels were small and near the limit of EDS sensitivity so this data is provided as qualitative. Elemental differences between the two groups showed slightly enriched calcium and a lack of sodium in the November diatoms while showing the complete opposite for the Cooper diatoms. A single fragment potentially from *Asterionella* or *Fragilaria* was found in the November sand from Tena Bar (Fig. 4B). This spectrum showed elevated levels of calcium and sodium again suggesting a difference from the *A. formosa* found on the Cooper bill which only showed enriched sodium. The single diatom spectrum from the March bill showed no increase in either sodium or calcium suggesting the March time frame has a different elemental abundance in the water from either the winter or Cooper sample suspected to have summer diatoms. The reproductive lifetime of a diatom is on the order of days^32^ so a difference in elemental abundance suggests that these three assemblages were from different seasonal periods.

## Conclusions

The mix of genera, abundance and elemental signatures suggests the Cooper bills did not get submerged during the November event but more closely aligns with a May–June time frame. Finding large diatoms on the bills rules out all theories involving human burial of dry bundles. This discounts the theories that the flight path was more westerly than the FBI chart shows and Cooper landed near and made his way along the Columbia River where he decided to bury some of the cash. It also discounts a perpetrator burying the money as a distraction at a later time. Finding summer diatoms rules out the theory that Cooper landed in the river in November, soaking the money and then buried some of it on shore. A summer time immersion and subsequent burial moves the money find completely away from the hijacking event in November. With the constraints put on the time of the submersion, many theories are easily discounted but as many things in the Cooper case, this new information does not bring forth any new theories on how or why the money would find its way to into the sand on Tena Bar during the summer months.

Diatoms have proven themselves as a forensic tool especially in the area of drowning and in certain cases where a local water body had uniquely identifiable species that were found on victims or evidence. Given the widespread abundance and tremendous species variation, combined with the robust silica frustule, diatoms are poised to make a significant contribution to forensic science. This is the first analysis using a diatom methodology of seasonal variation in population and species mix to time constrain a forensic event. The seasonal blooms of diatoms occur widely in water bodies all over the world but fortuitously the Columbia River has been well studied and these blooms seasonally documented. The elemental analysis shown here suggests that seasonal elemental differences might be particularly beneficial if found to hold true across different water bodies. The combination of species mix, abundance and trace elements holds the promise of diatoms as a statistically significant tool in constraining seasonal timelines involving water bodies.
